# Synthesis and Electrochemical Properties of Oleylamine as a Sour Saline Corrosion Inhibitor Under Laminar Flow at 40 °C

**DOI:** 10.3390/ma17215284

**Published:** 2024-10-30

**Authors:** Jorge Alvarez-Malpica, Karime Carrera-Gutiérrez, Manuel Chinchillas-Chinchillas, Manuel Herrera Zaldivar, Alfredo Martinez-Garcia, Victor M. Orozco-Carmona

**Affiliations:** 1 Centro de Investigación en Materiales Avanzados, Avenida Miguel de Cervantes Saavedra 120, Chihuahua 31136, Chihuahua, Mexico; alvarezj@imp.mx (J.A.-M.); karime.carrera@cimav.edu.mx (K.C.-G.); 2 Departamento de Ingeniería y Tecnología, Universidad Autónoma de Occidente (UAdeO), Blvd. Macario Gaxiola y Carretera Internacional, Los Mochis 81217, Sinaloa, Mexico; manuel.chinchillas@uadeo.mx; 3 Centro de Graduados e Investigación del Instituto Tecnológico de Tijuana, Blvd. Industrial, Tijuana 22444, Baja California, Mexico; zaldivar@ens.cnyn.unam.mx; 4 Departamento de Metalurgia e Integridad Estructural, Centro de Investigación en Materiales Avanzados, Avenida Miguel de Cervantes Saavedra 120, Chihuahua 31136, Chihuahua, Mexico

**Keywords:** sour corrosion inhibitor, oleylamine, impedance spectroscopy, inhibitor synthesis, laminar flow

## Abstract

In this study, the synthesis of a long-chain aliphatic amino compound and its sour corrosion inhibition properties were reported. Oleylamine was obtained through the reaction of 4-(Aminomethyl) pyridine with 1-chloro-octadecane. The identification and characterization of reaction products were carried out through Fourier transform infrared spectroscopy (FTIR) and gas chromatography/mass spectroscopy (GC-MS). Oleylamine was tested as a sour corrosion inhibitor for steels. Different concentrations of oleylamine (0, 5, 10, 25, and 100 ppm) in a sour saline electrolyte were analyzed. The dynamic anticorrosive behavior of oleylamine on carbon mild steel (AISI 1018) surfaces was evaluated using a laminar flow of 100 rpm and tested with potentiodynamic polarization (PDP) and electrochemical impedance spectroscopy (EIS) measurements. After electrochemical testing, the surface of the steel specimens that were used was characterized with Fourier transform infrared spectroscopy (FTIR) and scanning electron microscopy (SEM). The electrochemical results of the anticorrosive efficiency of oleylamine for steel showed an exponential behavior as a function of inhibitor concentration. At a concentration of 20 ppm of the inhibitor, the anticorrosive efficiency did not show any significant changes. However, at 100 ppm of the inhibitor, an efficiency of over 95% was achieved. After the electrochemical tests, the surface of the steel samples with the inhibitor revealed the formation of an inhibitor layer that prevented the corrosion of the steel.

## 1. Introduction

In oilfields, metallic pipelines and gathering tanks are commonly damaged by corrosion during production, transportation, storage, and processing. In pipeline systems, there is a simultaneous flow of crude oil, water (usually seawater), sand particles, and some gases, such as carbon dioxide (CO_2_) and hydrogen sulfide (H_2_S) [1,2]. It is well known that corrosion can be produced in several forms, among which CO_2_ corrosion (sweet corrosion), H_2_S corrosion (sour corrosion), and corrosion by oxygen dissolved in water are by far the most prevalent forms of attack found in oil and gas production [3]. Sour corrosion is defined as the corrosion of metals in an aqueous environment containing acidic gases (H_2_S/CO_2_) [4]. All impurities (chlorides and cyanide) have a certain influence on the corrosion of carbon steels, but H_2_S represents a longstanding problem in the oil and gas industry because it is very soluble in water; it is 200 times more soluble than oxygen and three times more soluble than CO_2_ in water at atmospheric pressure and temperature [5]. H_2_S interacts with steel surfaces to cause the formation of several iron sulfide phases, which results in pitting corrosion [6].

The most viable solution to this problem is injecting “special additives” into pipelines and tanks affected by sour environments. The use of corrosion inhibitors is one of the most economical and reliable approaches for controlling the corrosion of metallic structures in oil and gas facilities [7]. Once an inhibitor is adsorbed onto the metal surface, it can physically limit the interaction of molecules and ions with the surface, thus preventing corrosion. Many studies have been conducted to develop advanced inhibitor materials to withstand H_2_S corrosion at different temperatures and pressures.

Amines are derivatives of ammonia; their structure contains nitrogen atoms with an electron pair, and they are one of the groups of compounds that have improved inhibition efficiency against sour corrosion in carbon steel [8]. Corrosive compounds are inhibited by the adsorption of the amine group onto the metal surface through electrostatic interactions [8]. The oily layer formed by amine compounds on the steel surface works as a physical barrier to prevent sour corrosion; in an appropriate amount, these can protect the surface of the alloy at temperatures up to 177 °C [9]. Mok et al. reported that amine-based inhibitors provide up to 99% protection of steel in sour conditions [10].

Some of the most studied inhibitors were ethylenediamine, triethylenetetraamine, phenylenediamine, trimethylenediamine, *N*-aminoethylethanolamine, propylenediamine, methyliminobispropylamine, cyclohexylamine, 2-methylcyclohexylamine, and tetraethylenepentamine [2,11,12]. However, several of the compounds studied are aromatic and short-chain aliphatic compounds, and few studies have been carried out on large-chain aliphatic amino compounds (LCAAs). Some studies of aliphatic amino compounds showed better efficiency than that of the reported aromatic amino compounds [13,14,15,16]. For example, Yoshioka et al. studied the anticorrosive properties of oleylpropanediamine films on copper surfaces and observed better anticorrosive properties and lower toxicity [14]. Oleylamine films deposited on iron samples showed excellent anticorrosive properties in environments with sulfate, carbonate, and chloride ions [13].

The anticorrosive behavior of oleylamine films on surfaces of alloys such as carbon steel and Incoloy 800 used in pressurized water reactors for steam generation has also been studied [15,16]. However, there is a lack of information about the efficiency of long-chain aliphatic amino compounds as a sour corrosion inhibitor in carbon steel for their application in oilfields, oil/gas production, and fossil fuel production. Therefore, this study focuses on the evaluation of the electrochemical properties of an LCAA as a sour corrosion inhibitor in low-carbon mild steel (AISI 1018). Furthermore, in this work, the synthesis of an LCAA through nucleophilic substitution is proposed [17]. For this experiment, an alkyl halide compound is used as the substrate, and an aminomethyl-pyridine compound is used as the nucleophile source. The first step consists of a slow ionization of the substrate and the ionization of the alkyl halide to form the carbocation while assisted by a solvent [18]. The second step is a fast reaction between the carbocation and the nucleophile.

## 2. Materials and Methods

### 2.1. Synthesis and Characterization of the Sour Corrosion Inhibitor

**Figure 1:**
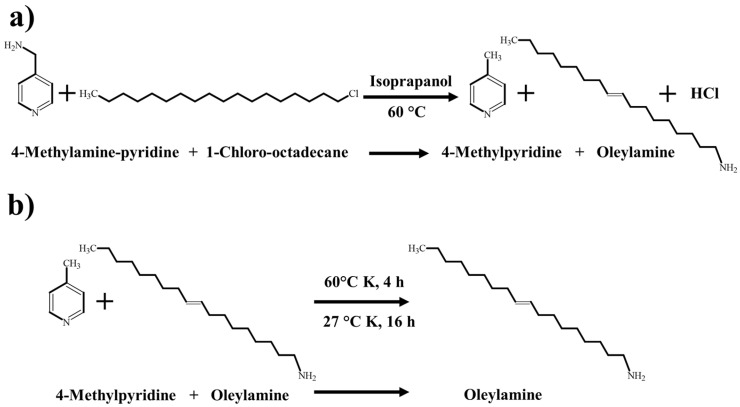
Route of oleylamine ((Z)-octadec-9-en-1-amine) synthesis starting from 4-methylamien-pyridine and 1-cloro-octadecane. (**a**) Formation of oleylamine and (**b**) Evaporation of byproducts and solvent.

All raw materials, solvents, and reagents used in the inhibitor synthesis were acquired from commercial suppliers directly and used as received. Starting from a stoichiometric mixture of 4-methylamino-pyridine (Sigma-Aldrich, St. Louis, MO, USA) and 1-chloro-octadecane (Sigma-Aldrich, St. Louis, MO, USA), oleylamine was synthesized as a sour corrosion inhibitor. The reagents were mixed and solubilized in isopropanol; then, the reaction was carried out in a ball reactor at 60 °C with constant stirring until the reaction was completed. The reaction of the precursors was monitored using thin-layer chromatography (Figure 1a). Once the reaction was finished, the reaction product was heated to 60 °C and subjected to constant stirring for 4 h until the evaporation of the organic solvents and other compounds formed during the reaction occurred (Figure 1b). Finally, the corrosion inhibitor was aged at 27 °C for 16 h (Figure 1b).

#### 2.1.1. Fourier Transform Infrared Spectroscopy with Attenuated Total Reflection (FTIR-ATR)

Infrared spectra were obtained from a standard Shimadzu IRAffinity-1S infrared spectrometer equipped with an attenuated total reflection crystal. The characterization was performed by scanning the mid- and near-infrared spectrum (4000–400 cm^−1^).

#### 2.1.2. Gas Chromatography–Mass Spectroscopy (GC-MS)

A gas chromatography–mass spectroscopy analysis was performed on a GCMS-QP2010 SE instrument (Shimadzu, Tokio, Japan) equipped with an Inert Cap WAX-HT capillary column (30 m × 0.25 mm i.d., 0.25 µm). The operating conditions were a split ratio of 50:1 and a temperature of 300 °C. Methylene chloride was used as a solvent to prepare the inhibitor sample.

### 2.2. Preparation of Steel Cylinders as Working Electrodes

Low-carbon mild steel (AISI 1018) with a composition of C (0.14%), P (0.04%), Mn (0.41%), P (0.04%), Si (0.15%), S (0.05%), and Fe (balanced) was used as a working electrode (WE). Cylindrical steel specimens had a length of 1.2 cm and a diameter of 0.8 cm. The surface of each steel specimen was cleaned according to the ASTM standard procedure G1-03 [19]. In brief, each WE surface was ground with silicon carbide sheets with a grade ranging from 400 to 1200, followed by thorough washing with double-distilled water. Later, the samples were sonicated in ethanol and, finally, dried under warm air.

### 2.3. Preparation of the Sour Saline Electrolyte

The sour saline electrolyte was a H_2_S-saturated saline solution created according to NACE ID182; it was prepared with double-distilled water and contained 3.5 wt % NaCl, 0.305 wt % CaCl_2_·2H_2_O, and 0.186 wt % MgCl_2_·6H_2_O [20]. The saline solution was saturated with H_2_S until reaching a concentration of 600 ± 50 ppm. The concentration of H_2_S in the sour electrolyte was determined through volumetric chemical analysis (iodometric titration).

### 2.4. Setup of the Electrochemical Cell

**Figure 2:**
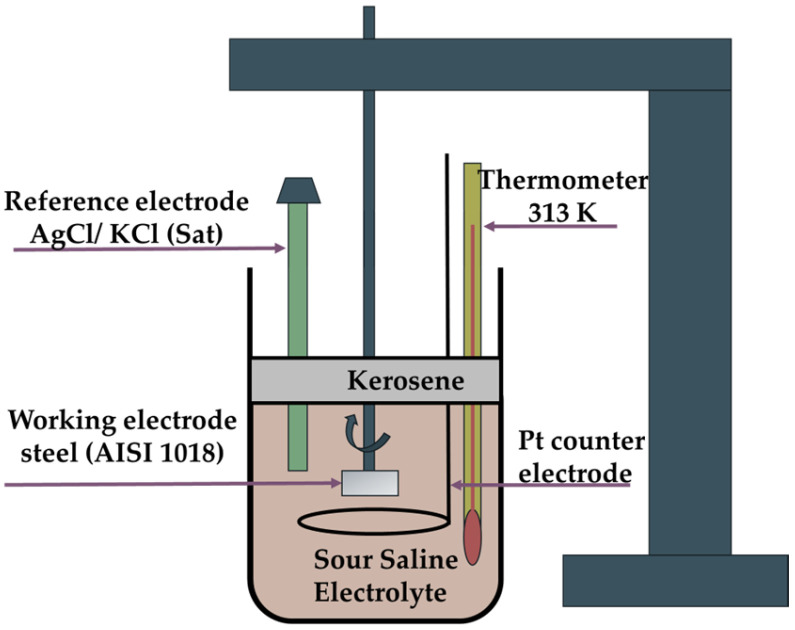
Diagram of the electrochemical cell setup.

The electrochemical cell was filled with 500 mL of sour saline electrolyte and 100 mL of low-odor kerosene (P339-01, Baker, Madrid, Spain), which was used as a lid layer. The kerosene seal was used to prevent the evaporation of H_2_S from the electrolyte. The electrodes of the system were immersed in the sour saline electrolyte, and subsequently, the corrosion inhibitor was injected into the electrolyte at concentrations of 5, 10, 50, and 100 ppm. Prior to this, the kerosene seal was collocated (Figure 2). Finally, the working electrode was connected to a ring–disk system composed of a rotating cylinder (PAR 636-A) controlled by a speed rotator (Princeton Applied Research model 636).

### 2.5. Electrochemical Measurement

All electrochemical tests were performed in a potentiostat/galvanostat (Autolab PGSTAT 100 (Metrohm, Herisau, Switzerland)). Electrochemical experiments were conducted using a standard open three-electrode glass cell consisting of a platinum counter electrode (CE), an Ag/AgCl (KCl sat) reference electrode (RE), and steel cylinders as working electrodes (WEs). Potentiodynamic polarization (PDP) tests were performed at ±1.0 V vs. OCP using a scan rate of 10 mV/s [21]. Electrochemical impedance spectroscopy (EIS) was carried out in the frequency range of 100,000–0.01 Hz with a voltage amplitude of 10 mV according to ASTM D8370-22. The experiments were performed at 40 °C in the cell. Each electrochemical measurement was carried out in hydrodynamic conditions with a rotation speed of 100 rpm (0.042 m/s) to achieve lamellar flux, and the temperature was held to 40 °C using a temperature plate. Corrosion current densities (*I_corr_*), corrosion potentials (*E_corr_*), and the impedance equivalent circuits were calculated using NOVA 2.1.7 software (Metrohm, Switzerland).

### 2.6. Characterization of the Steel Surface After Electrochemical Tests Using Scanning Electron Microscopy (SEM)

The surface of low-carbon mild steel specimens with and without inhibitors was examined using scanning electron microscopy (FHitachi SU3500 10 kV, Tokyo, Japan). The analysis was conducted under an accelerating voltage of 10–15 kV in the secondary electron mode.

## 3. Results

### 3.1. Corrosion Inhibitor Synthesis

**Figure 3:**
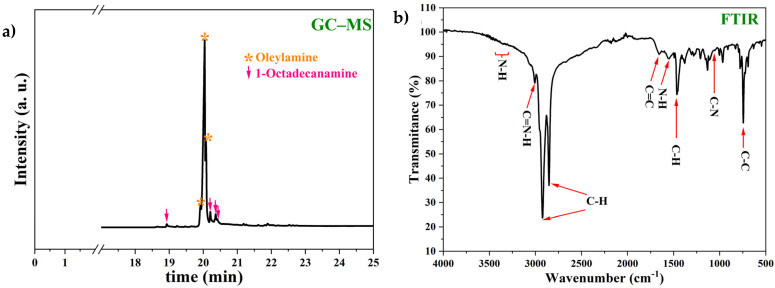
(**a**) GC-MS and (**b**) FTIR-ATR analyses of the product obtained from the reaction of 4-(aminomethyl) pyridine with 1-chloro-octadecane.

The product forming from the reaction of 4-(aminomethyl)pyridine with 1-chloro-octadecane was identified and characterized using gas chromatography–mass spectroscopy (GC-MS) and Fourier transform infrared spectroscopy (FTIR), as shown in Figure 3.

In the GC-MS spectrum shown in Figure 3a, the characteristic signals of oleylamine (orange asterisk) and the presence of other peaks associated with the typical signals of 1-octadecylamine (pink arrow) were identified using the Chemical Abstracts Service database (CAS_112-90-3 and CAS_124-30-1). The identification of both compounds suggested that the inhibitor was a mixture of oleylamine and octadecylamine, but due to the area of the peaks in the spectrum, 1-octadecylamine was found at a low concentration [22]. On the other hand, based on the Chemical Abstracts Service database (CAS_112-90-3), it was possible to identify the formation of oleylamine in the reaction products with the FTIR-ATR spectrum shown in Figure 3b. The characteristic bands of oleylamine in the FTIR-ATR spectrum shown in Figure 3b were in agreement with the oleylamine bands reported in the literature; the bands at 3380–348 and 1550 cm^−1^ corresponded to N–H bonds, the bands at 3007 and 1053 cm^−1^ were attributed to C–N–H and C–N bonds, the bands at 2920–2852 and 1150 cm^−1^ were due to C–H bonds, and the bands at 1651 and 774 cm^−1^ were associated with C=C and C–C bonds, respectively [23,24,25,26]. In the FTIR spectrum, the characteristic bands of octadecylamine were not observed, so it could be interpreted that the octadecylamine concentration in the inhibitor was low.

In addition to the identification of the reaction product, the reaction yield was calculated using Equation (1) [27]. The oleylamine synthesis was carried out in triplicate, and a reaction yield of 91.23% was reached. Therefore, the reaction presented excellent efficiency.

(1) $$Yield\left(\%\right)=\frac{amount\;of\;experimental\;reaction\;product}{amount\;of\;theorical\;reaction\;product}$$

### 3.2. Electrochemical Properties of the Inhibitor

**Figure 4:**
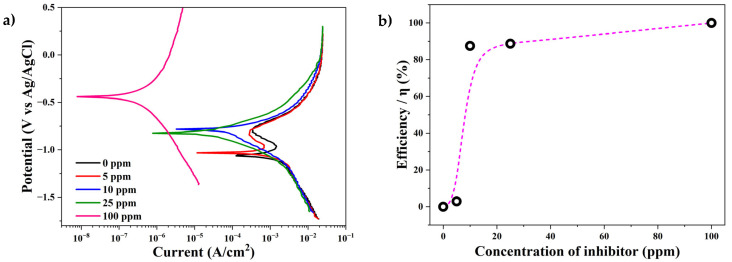
(**a**) Potentiodynamic polarization curves and (**b**) inhibition efficiency of oleylamine vs. the inhibitor concentration curve in the presence of sour saline electrolytes for AISI 1018 mild steel at 40 °C obtained through PDP.

Figure 4 shows the effects of inhibitor concentration on the anodic and cathodic polarization curves for low-carbon mild steel (AISI 1018) in an H_2_S-saturated saline solution at 40 °C. In the polarization curves of the non-inhibited electrolyte (0 ppm), it can be observed that the anodic region was controlled by the activation process, while the cathodic region was governed by diffusion (black line). In addition, the increase and decrease in the corrosion current in the anodic region revealed the formation of a passivation layer. It has been reported that the passivation layer in a working electrode is caused by the formation of an iron (FeS) sulfide layer due to the interaction of steel surfaces with H_2_S in sour solutions, as described by the following equation (Equation (2)) [5,28].

(2) $$Fe+S_{2}H\;\to FeS+H_{2}$$

In Figure 4a, the inhibitor concentration increases in the electrolyte led to a shift in the E_corr_ values to more positive values. This was an indication that the anodic process of AISI 1018 mild steel was inhibited by the corrosion inhibitors. In addition, a decrease in the corrosion current density in both the cathodic and anodic branches of the curves indicated a decrease in the interaction of electrolyte compounds with steel due to the formation of an inhibitor layer on the steel surface. The changes in the corrosion current of the anodic region due to the presence of the inhibitor indicated that the formation of the passivation layer was affected (until 5 ppm). For inhibitor concentrations of >10 ppm in the electrolyte, the passivation layer was not detected.

The potentiodynamic polarization plots in Figure 4a were utilized to determine the corrosion parameters for the mild steel exposed to H_2_S-saturated saline solution with and without the inhibitor. The corrosion potential (*E_corr_*), corrosion current density (*I_corr_*), and corrosion rate data are summarized in Table 1. There was a decrease in *E_corr_*, *I_corr_*, and the corrosion rate with the increase in the inhibitor concentration. For a concentration of 100 ppm, *I_corr_* decreased from 352.0 µA/cm^2^ (without inhibitor) to 0.04 µA/cm^2^, and the corrosion rate (mm/year) decreased from 4.08 to 4.64 × 10^−4^. Additionally, the inhibitor efficiency was calculated through Equation (3) [2].

(3) $$\eta =\frac{I_{corr}-I_{corr}^{0}}{I_{corr}}\times 100$$

Here, *I_corr_* is the corrosion current without the inhibitor, and *I^0^_corr_* is the corrosion current with the inhibitor. According to the values reported in Table 1, 100 ppm of inhibitor in the electrolyte achieved 99.98% efficiency as an H_2_S corrosion inhibitor. On the other hand, Figure 3b reveals that the inhibitor showed exponential behavior; with this, it can be argued that after a concentration of 10 ppm of inhibitor, the increase in efficiency was less steep.

**Figure 5:**
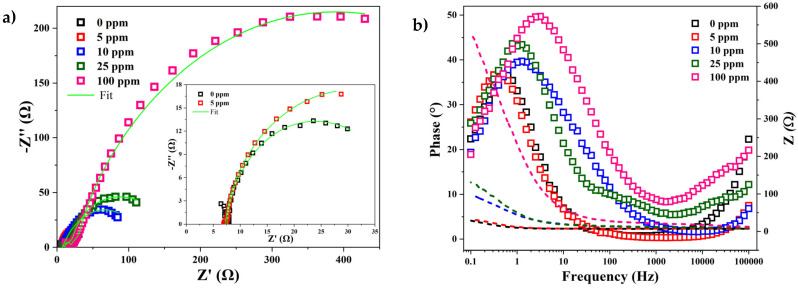
(**a**) Nyquist diagram and (**b**) Bode diagram (phase angle–impedance) of steel specimens immersed in the H_2_S saline electrolyte at 40 °C with and without the inhibitor.

The impedance spectra of low-carbon mild steel with and without the inhibitor in a sour saline electrolyte are shown in Figure 5. The Nyquist and Bode plots (Figure 5a,b) show the influence of the inhibitor content on the corrosion of the steel surface. The shapes of the spectra in the Nyquist plots were very similar in the experiments with and without the inhibitor, but as the inhibitor content increased, the width of the semicircle increased (Figure 5a). Moreover, in the case of the Bode plot in the phase angle format (square), a higher phase angle was observed with the increase in the amount of inhibitor in the electrolyte (Figure 5b). Studies reported that a higher phase angle is associated with a reduction in the interaction between Fe and hydrogen diffusion processes due to the formation of a more protective inhibitor film [29]. However, in the Bode graph, where the impedance was a function of frequency (dotted lines), it can be observed that the higher the inhibitor content, the higher the required frequency. This can be interpreted as a greater resistance to charge transfer.

**Figure 6:**
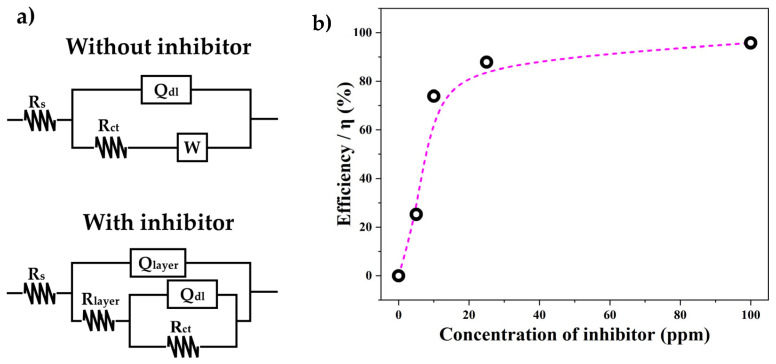
(**a**) Equivalent circuit used to fit the EIS spectra obtained without and with the inhibitor. (**b**) Curve of oleylamine inhibitor efficiency vs. concentration of the inhibitor in a sour saline electrolyte for AISI 1018 mild steel at 40 °C obtained through EIS.

The proposed equivalent circuits for fitting the experimental impedance diagrams under dynamic conditions are shown in Figure 6a. For the impedance plots without the inhibitor, an equivalent circuit consisting of a resistor *R_s_* connected in series to a constant phase element (*Q_dl_*) parallel to a resistor (*R_ct_*) that is connected in series to a Warburg-like impedance element (*W*) is proposed. Here, *R_s_* corresponds to the resistance of the electrolyte to the current, *R_ct_* is the resistance to charge transfer at the interface between the electrolyte and the steel surface, the constant phase element *Q_dl_* is associated with the electrochemical double layer formed between the electrolyte interface and the steel surface, and finally, there is a Warburg-like impedance element (*W*) related to the hydrogen semi-infinite diffusion on the hydrogen sulfur scale [30]. This equivalent circuit arrangement is typical of steel corrosion processes in NaCl (30 g/L) and H_2_S (100–2250 ppm) solutions, which involve diffusion processes and iron sulfide formation [30]. For the impedance plots with the inhibitor, an equivalent circuit consisting of a resistance *R_s_* connected in series to a constant phase element (*Q_layer_*) parallel to a resistance (*R_layer_*) connected in series to a constant phase element (*Q_dl_*) that is parallel to a resistance (*R_ct_*) is proposed. Here, R_s_ corresponds to the resistance of the electrolyte to the current, *R_layer_* is the resistance of the inhibitor layer to the current, *R_ct_* is the charge transfer resistance between anodic and cathodic zones of the steel surface, and the constant phase elements *Q_layer_* and *Q_dl_* are associated with the layer formed by the inhibitor on the steel surface and the electrochemical double layer formed between the interface of the inhibitor and the steel surface, respectively [28]. This circuit was proposed to fit the corrosion mechanism in alkaline sour environments (7.0 < pH < 8.8) [31,32,33]. Therefore, it could be assumed that the addition of the inhibitor in the electrolyte promoted an alkaline environment. The values of electrolyte resistance (*R_s_*), the inhibitor layer’s resistance on the steel surface (*R_layer_*), and the charge transfer resistance in the electrochemical double layer (*R_ct_*) are summarized in Table 2. It can be observed that the *R_s_* value of the electrolyte for the blank sample was 7.6 Ω, and the charge transfer resistance of the steel surface (*R_ct_*) was 31.2 Ω. However, in the samples with the inhibitor, the inhibitor layer showed an *R_layer_* value that increased from 0.98 to 14.6 Ω with the increase in the concentration of 5 to 100 ppm of inhibitor. Some authors have observed that an increase in the *R_layer_* value is associated with the thickness of the inhibitor layer [33]. On the other hand, the *R_ct_* value in the electrochemical double layer increased from 31.2 Ω without the inhibitor to 724 Ω in the samples with 100 ppm of inhibitor. This showed that the inhibitor reduced the electron transfer between the electrolyte and the steel surface, resulting in a reduced corrosion rate of the steel. The values of *R_layer_* and *R_ct_* were summed to obtain the total resistance (R) of the inhibitor, and the corrosion efficiency of oleylamine as a sour saline corrosion inhibitor was calculated using Equation (4) [28,33].

(4) $$\eta =\frac{R-R^{0}}{R}\times 100$$

Here, η is the efficiency, and *R* and *R*^0^ are the total resistance with and without the inhibitor, respectively. The efficiency values are summarized in Table 2 and plotted in Figure 6b. It can be observed that the corrosion efficiency of the inhibitor obtained with *I_corr_* (Figure 4b) and *R_ct_* (Figure 6b) showed the same behavior (exponential curve), but with the impedance results, a better fit to the exponential curve was obtained. The inflection point of the corrosion efficiency curve was observed near 20 ppm of inhibitor. After this concentration, there were no significant changes in the inhibition efficiency. The maximum efficiency (95.77%) was reached at a concentration of 100 ppm of inhibitor.

### 3.3. Characterization of Cylindrical Mild Steel Surface After Electrochemical Tests

**Figure 7:**
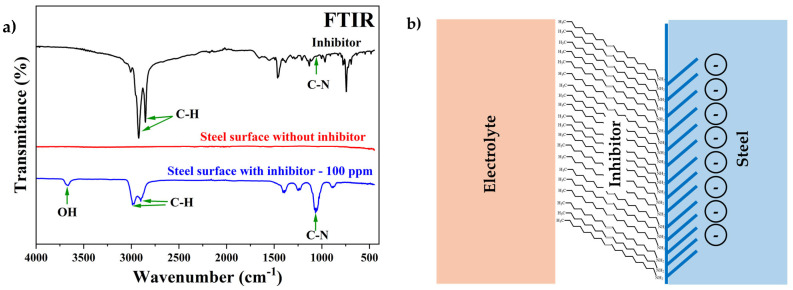
(**a**) FTIR spectrum of the steel surface with and without inhibitor after electrochemical tests. (**b**) Diagram of the interaction of the inhibitor with the steel surface and electrolyte.

The cylindrical steel specimen surface with and without the inhibitor was characterized using FTIR, as shown in Figure 7a. On the steel specimen surface without the inhibitor, the presence of no compounds was detected, but on the steel sample with 100 ppm of inhibitor, C–H, C–N, and OH bands were observed in the FTIR spectrum. Although the FTIR spectrum was different from the inhibitor’s spectrum (before and after the electrochemical test), some functional groups with slight shifts could be identified, such as C–H (2984 and 2904 cm^−1^), OH (3663 cm^−1^), and C–N (1072 cm^−1^) [23,25]. These shifts have been studied by others in the literature, and it was suggested that they could be caused by the formation of an oleylamine layer covering the metal surface [26]. Some authors reported that the lone pair of electrons on the nitrogen atom can be donated to the vacant d-orbitals on the iron (Fe) atoms to form coordinated covalent bonds between the inhibitors and the metal surface [34]. Therefore, with this information, a diagram of how the inhibitor interacts with the steel and electrolyte was constructed, where the amino group of oleylamine is connected to the metal surface and the aliphatic chain is exposed to the electrolyte (Figure 7b).

**Figure 8:**
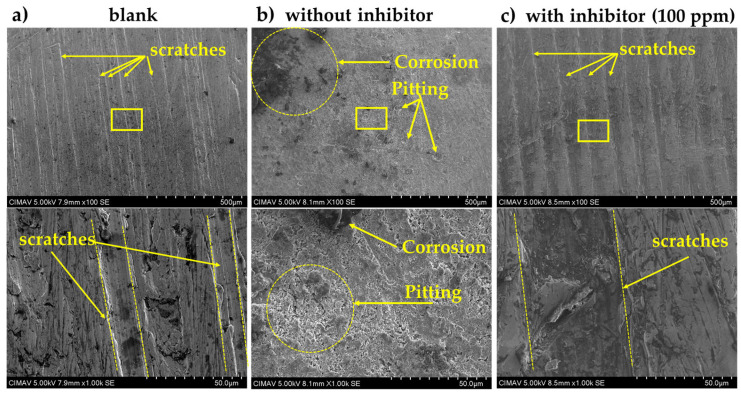
SEM micrographs of samples (**a**) unexposed to the sour saline electrolyte (blank), (**b**) exposed to the sour saline electrolyte without the inhibitor (0 ppm), and (**c**) exposed to the sour saline electrolyte with the inhibitor (100 ppm).

In addition, the surface morphology of low-carbon mild steel (AISI 1018) samples that were exposed and unexposed (blank) to the sour saline electrolyte with (100 ppm) and without (0 ppm) the inhibitor at 40 °C was examined through scanning electron microscopy; see Figure 8. The surface of mild steel that was not exposed to the sour saline electrolyte presented scratch marks due to sanding from the polishing treatment and did not show any corroded areas (Figure 8a). However, the sample exposed to the sour saline electrolyte without the inhibitor showed severe corrosion areas and corrosion pitting under hydrodynamic conditions. In addition, scratches caused by polishing were not observed, and this could be interpreted as surface wear due to electropolishing processes (Figure 8b). In the case of the mild steel surface exposed to the electrolyte with the inhibitor, no corrosion attacks or pitting were observed, the sample surface did not change, and the same morphology as that of the blank was shown (Figure 8c). The steel surface observed in the micrographs (Figure 8) and the results of FTIR and electrochemical tests (Figure 4, Figure 5 and Figure 7) showed the same results. Corrosion inhibition studies conducted under static and hydrodynamic conditions have shown excellent results [28]. Azghandi et al. used an acrylic terpolymer of methyl methacrylate/butyl acrylate/acrylic acid in a ratio of 49:49:2 wt % containing 50 wt % of water as a corrosion inhibitor in a simulated sour petroleum solution and reported good results under static conditions, but under hydrodynamic conditions, the steel surface showed corroded areas and pitting [26]. Therefore, the use of oleylamine as an inhibitor showed better performance under hydrodynamic conditions than that reported in the literature.

## 4. Conclusions

In this study, the successful synthesis of oleylamine using 4-(aminomethyl)pyridine with 1-chloro-octadecane and its electrochemical properties as a corrosion inhibitor in a sour (H_2_S) saline electrolyte were reported. The following main conclusions can be drawn.

(1) Through the reaction route proposed in this report, the oleylamine synthesis reached an excellent reaction yield of 91.23%.
(2) On the other hand, the electrochemical tests revealed that oleylamine possesses anticorrosive properties as an inhibitor when used on low-carbon mild steel (AISI 1018) surfaces exposed to sour saline electrolytes. According to the *I_corr_* and *R_ct_* parameters for the inhibitor concentration in the electrolyte, the efficiency of oleylamine as a corrosion inhibitor has exponential behavior.
(3) The application of 20 ppm of the inhibitor in the electrolyte reached an inhibition efficiency of more than 85%, and a maximum efficiency higher than 95% was reached at 100 ppm.
(4) Furthermore, SEM analysis confirmed the anticorrosive properties of oleylamine in steels exposed to sour electrolytes. In addition, oleylamine may be an excellent candidate as a corrosion inhibitor for application in sour saline environments at temperatures ≥40 °C due to its high boiling point (364 °C).

To broaden the application scenarios of oleylamine using 4-(aminomethyl)pyridine with 1-chloro-octadecane as a corrosion inhibitor, it is necessary to conduct further in-depth research with technological tests, applying the inhibitor in ducts that transport fluids with real solutions at different temperatures, pressures, etc.

## Figures and Tables

**Table 1:** Polarization parameter for steel specimens immersed in the H_2_S saline electrolyte (40 °C) vs. the concentration of the inhibitor.

Inhibitor Concentration	Corrosion Potential (Ecorr vs. Ag/AgCl)	Corrosion Current (Icorr)	Corrosion Rate	Efficiency η
ppm	V	µA/cm^2^	mm/year	%
0	−1.059	352.0	4.08	0
5	−1.028	341.8	3.96	2.94
10	−0.816	43.8	0.51	87.50
25	−0.800	39.8	0.46	88.72
100	−0.452	0.04	4.64 × 10^−4^	99.98

**Table 2:** Electrochemical impedance spectroscopy parameters for steel specimens immersed in the sour saline electrolyte (40 °C) while incorporating the inhibitor in different concentrations.

Sample	*R_s_*	*R_layer_*	*R_ct_*	Efficiency/*η*
Units	Ω	Ω	Ω	%
Blank	7.6	---	31.2	0
5 ppm	7.2	0.98	39.8	25.26
10 ppm	7.8	1.95	118.4	73.86
25 ppm	6.7	3.67	253.3	87.85
100 ppm	7.8	14.6	724.0	95.77

## Data Availability

All data generated or analyzed during this study are included in this published article.
